# AB103, a CD28 antagonist peptide: a new therapeutic agent in a model of severe sepsis

**DOI:** 10.1186/cc10404

**Published:** 2011-10-27

**Authors:** D Teleman, C-S Chung, A Ayala, S Opal, A Chahin, A Shirvan

**Affiliations:** 1 Atox Bio, Ness Ziona, Israel; 2 Alpert Medical School at Brown University, Rhode Island Hospital, Providence, RI, USA; 3 Memorial Hospital of Rhode Island, Pawtucket, RI, USA

## Introduction

AB103 is a novel CD28 antagonist peptide currently in clinical development that modulates CD28 signaling in T cells, without affecting the normal humoral immune response. In experimental models of Gram-positive, Gram-negative and polymicrobial sepsis, AB103 demonstrated significant activity, increasing overall survival.

## Methods

The AB103 activity and mode of action (MOA) were evaluated in a murine model of cecal ligation and puncture (CLP). AB103 (5 mg/kg) was administered to mice (Balb/c) at various times points following CLP (2 to 24 hours), together with moxifloxacin.

## Results

A single dose of AB103, given at 12 or 24 hours post CLP, rescued 100% and 62.2% of the animals (respectively) from sepsis-induced mortality, whereas moxifloxacin alone (LD_90_, given at 12 hours) rescued only 25% (*P *< 0.05) of the animals. In a separate set of experiments investigating the MOA, AB103 administration (5 mg/kg, given without antibiotics 2 hours post CLP) was associated with: a reduction in Th-1 cytokine levels in peritoneum (TNFα, IL-3, IL-17 and Rantes) and plasma (IL-3 and IL-6); a reduction in splenocyte proliferation, stimulated *ex vivo *with anti-CD3 and anti-CD28 antibodies; a reduction in neutrophil recruitment to the spleen, liver and kidney, as determined by MPO activity; and a reduced bacterial load in peritoneum, blood and tissues (kidney, liver, spleen).

## Conclusion

These data demonstrate that attenuation of CD28 signaling is a viable therapeutic approach to the treatment of sepsis. Due to its robust activity and good safety profile in humans already established in a phase 1 study, AB103 should be clinically evaluated in sepsis patients.

